# Development of a customizable interactions questionnaire (CIQ) for evaluating interactions with objects in augmented/virtual reality

**DOI:** 10.1007/s10055-022-00678-8

**Published:** 2022-08-25

**Authors:** Meiyuzi Gao, Deborah A. Boehm-Davis

**Affiliations:** Meta, Redmond, Washington USA

**Keywords:** Usability testing, Questionnaire, Metrics, User studies

## Abstract

As new methods for interacting with systems are being developed for use within augmented or virtual reality, their impact on the quality of the user’s experience needs to be assessed. Although many instruments exist for evaluating the overall user experience or the computer interface used to complete tasks, few provide measures that can be used to evaluate the specific forms of interaction typically used in these environments. This paper describes the development of a customizable questionnaire for measuring the subjective user experience that focuses on the quality of the interactions with objects in augmented reality/virtual reality (AR/VR) worlds, which we are calling the Customizable Interactions Questionnaire, or (CIQ). The final questionnaire measures five factors that are related to user satisfaction while using the system: quality of interactions, assessment of task performance, comfort, quality of sensory enhancements, and consistency with expectations.

## Introduction

As virtual and augmented reality systems are maturing, new methods for interacting with these systems are also being developed. As these new interaction methods are developed, they should be evaluated. The assessed quality of the interactions then can be used to (a) define the minimal level of quality considered acceptable for an input device to be viable; or (b) compare the quality of interaction delivered by a proposed or new device relative to a benchmark or competing system.

There are many studies evaluating specific input devices (see, for example, Dickie et al. 2006; MacKenzie and Jusoh 2001; MacKenzie et al. 1991) in the general HCI literature. In addition, there are ISO standards that have been developed (ISO 2008, 2012) and literature providing guidance on how to evaluate the quality of input devices using representative tasks, models of performance, or taxonomies (Wanderley and Orio 2002). However, there are fewer studies that specifically address the forms of interaction used in AR/VR settings (see, for example, Fahmi et al. 2020; Whitmire et al. 2018). Many of these empirical studies have employed a questionnaire as an adjunct to the collection of quantitative data. However, these questionnaires have been specific to the devices they evaluated and across these studies, they do not employ common questions.

Our goal for this work was to develop a questionnaire that is psychometrically valid, that can be customized for specific input devices, and that is useful in evaluating the quality of interactions with objects in virtual or augmented reality. Many instruments exist for evaluating the overall user experience, the computer interface or the content displayed to users (e.g., Brooke 1996; Chin et al. 1987; Lewis 1995; Sauro and Dumas 2009), or for evaluating the overall experience of presence in an environment (e.g., Lessiter et al 2001; Witmer and Singer 1998). They have been used in virtual and augmented reality (AR/VR) research to assess the constructs of overall user experience such as usability, workload, and frustration, e.g., Lu et al. 2020. However, these instruments are not suitable for evaluating new interactions methods developed for AR and VR (see, e.g., Haptx 2019; Karev 2018; Tactical haptics 2019) because they are designed to assess the overall experience.

In assessing interactions in a virtual or augmented world, it is important to remember that different features may need to be assessed than when evaluating an input device to a two-dimensional computer screen. Virtual and augmented worlds allow users to interact with both virtual and real objects. These interactions will often happen through use of the hands. Thus, evaluating the ease with which this can be done will need to include an evaluative component that assesses, for example, the quality of manual interaction as users interact with objects in the environment. It may also be important to evaluate the extent to which users find the interactions with objects “believable” (Cai 2016; Samad et al. 2019; Suchoski et al. 2016); that is, interactions that are not consistent with a user’s previous experience may degrade the quality of a user’s experience in manipulating objects.

Although some instruments allow researchers to assess interaction level components within an AR/VR experience (e.g., the Virtual Embodiment Questionnaire, Roth and Latoschik 2019), they are not sufficiently flexible to assess emergent interaction designs in AR/VR environments that are created with specific hardware input devices or targeted at specific use cases. Further, few existing measures allow their question stems to be tailored or customized. This work developed a questionnaire that can be customized to new interaction methods as a solution to the challenges stated above. The questionnaire measures subjective reactions of users to specific modes of interaction used in VR and AR applications. The questionnaire was validated through psychometric analyses for its reliability and sensitivity in VR testing. An initial exploration of its use in AR was also conducted. This questionnaire will allow researchers to use a psychometrically valid instrument when evaluating new forms of interaction in VR, and potentially, AR.

## Overview of our approach

This section provides a description of our overall approach to developing and validating the questionnaire. An initial literature review was used to identify potential constructs that might be related to the quality of manual interactions with virtual objects in augmented/virtual worlds. Following this, interviews were conducted with members of our research team to identify potential additional constructs. It is possible that these experts in the use of AR/VR devices may have identified constructs that are only relevant to our devices. However, given that our experiments used off-the-shelf games as a platform, any suggestions they made that were not more generally relevant should have fallen away in the factor analyses conducted on the data.

The constructs identified were wearability/encumbrance of the device, comfort, perceived system responsiveness, extent to which sensory characteristics enhanced the interaction, object believability, limb ownership, cognitive load, and learnability. Questions designed to measure these constructs were drawn from several sources. One source of questions for measuring these constructs was existing questionnaires, e.g., Brooke 1996; Chin et al. 1987; Hart and Staveland 1988; Lewis 1995; Paas et al. 1994; Sauro and Dumas 2009; Slater et al. 1995. Another source was questions previously used and validated by our team in measuring the dimensions of interest. From that merged list of questions, we identified the smallest set of questions that we felt would provide us with information on each construct without duplication as the starting point for our list of questions. The twenty-one questions selected were revised following the advice presented in Vannette (2019) which describes best practice in developing questionnaire item stems and response anchors.

The initial set of questions generated were examined in a cognitive interview study, following the guidance outlined in Willis (1999). The cognitive interviews yielded several suggestions for changes to the question stems which were implemented prior to the conduct of the full validation studies.

The first study was designed to validate the questionnaire through both in-laboratory and remote (online) data collections. Given our interest in evaluating manual interactions in a virtual environment, we asked users to evaluate one or more VR games that represented the use of hands in different ways, some of which were more consistent with the use of our hands than others. All games ran on the Oculus Quest and used the same method of tracking the users’ real hands. Participants in the laboratory evaluated each game after playing it; users online evaluated a game only if they had played it in the past week.

The validation process was repeated in two additional studies that took place 8 months apart. One study applied a similar experimental design with the addition of five new questions based on subject matter experts’ feedback, conducted as an online experiment. The second study, also online, adapted the questionnaire to augmented reality (AR) environments to explore the extent to which the questionnaire could be used in that setting in addition to the VR environment in which it was initially validated.

## Cognitive interview study

The goal of the cognitive interview study was to evaluate how people understood and responded to the questions in the first draft of the proposed questionnaire. Recommended practice in developing a questionnaire is to ask participants how they interpret the stem portion of each question. This allows the researchers to refine the language used in the question stems to ensure that all participants responding to the final questionnaire interpret the question in the same way. Participants with limited exposure to VR were chosen for this study as the purpose of the cognitive interview was to evaluate the comprehensibility and accessibility of the wording such that people without extensive experience in the technical space would be able to understand and answer the questions. The results from this study were leveraged to improve the quality of question stems and response alternatives in the questionnaire.

### Participants

Four male and four female native English speakers between 26 and 65 years were recruited in the Pacific Northwestern region of the USA. Six of the participants had completed a college degree; the remaining two had some exposure to college education. Seven of the eight participants had little or no prior experience with virtual reality technology before coming to the study; one participant reported using VR once a month.

### Materials

This study was held in a quiet room with a recording device installed to capture audio content. Materials included a consent form, a standard Oculus Quest VR headset with two controllers, and the draft questionnaire.

### Procedure

Each session lasted between 60 and 90 min. One participant was interviewed per session; participants were compensated for their participation.

Upon arrival, participants indicated their intention to participate by signing the consent form and completing a demographic questionnaire. They then engaged in a tutorial application that demonstrates hand-based interactions in VR (First Steps, 2019) using an Oculus Quest headset and touch controllers for the next 10–20 min. After completing the demonstration, participants examined the items in the questionnaire one by one. For each item, participants reported their answer. They were also prompted by the researcher to share their interpretation of the question stem and their decision-making process. Examples of prompts were “Can you repeat this question in your own words?”, “What is [phrase] to you?” or “How did you arrive at this answer?” Once participants had addressed every question, they were asked to review the questionnaire holistically for additional comments and feedback. Lastly, participants were debriefed and thanked.

### Results and discussion

Participants reported inconsistent responses or evaluations in 10 out of 21 questions. Our focus was on identifying places where the participants had differing interpretations of the specific wording found in individual questions. This is the crux of the purpose of the cognitive interview approach. If participants interpret the specific words used in the question stem in different ways, it will not be possible to get reliable responses across participants with the final questionnaire. That is, to the extent that participants interpret a word differently, they are responding to a different question, making interpretation of their responses impossible.

In each case identified, we edited the wording of our questions to consider the different interpretations caused by the original wording in our questions. For example, when asked “To what extent did the virtual hands feel like your hands?”, participants used different criteria in their evaluation (e.g., inconsistency of hand gestures; consistency of hand locations; how difficult it was to learn how to interact with objects; or size of the virtual hand). As another example, when asked “How fast or slow did the demo respond to your actions?”, participants found that the word “demo” was not sufficiently specific for them to be clear about what was meant. In all cases where confusion was identified, changes were made to the survey. In the first example cited, the question stem was not changed, but an additional question was asked to disambiguate the source of the rating. Specifically, when the participant assigned a rating greater than or less than 4 (indicating the virtual hands did, or did not, feel like their hands), they were provided with choices indicating why they felt this way, to allow us to disambiguate the basis of their rating. In the second example given, the question was changed to “How fast or slow did the *virtual world* respond to your actions?” This was done to clarify the aspect of the demo that we wanted them to evaluate.

## Psychometric validation studies

**Fig. 1:**
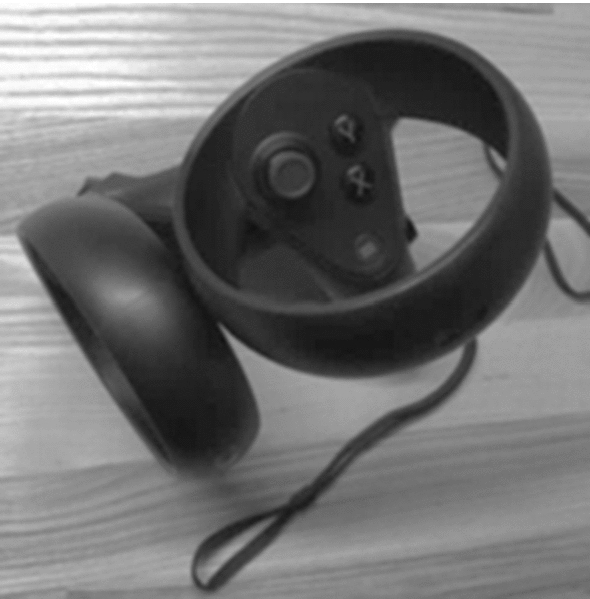
The Oculus controllers used in the studies

**Fig. 2:**
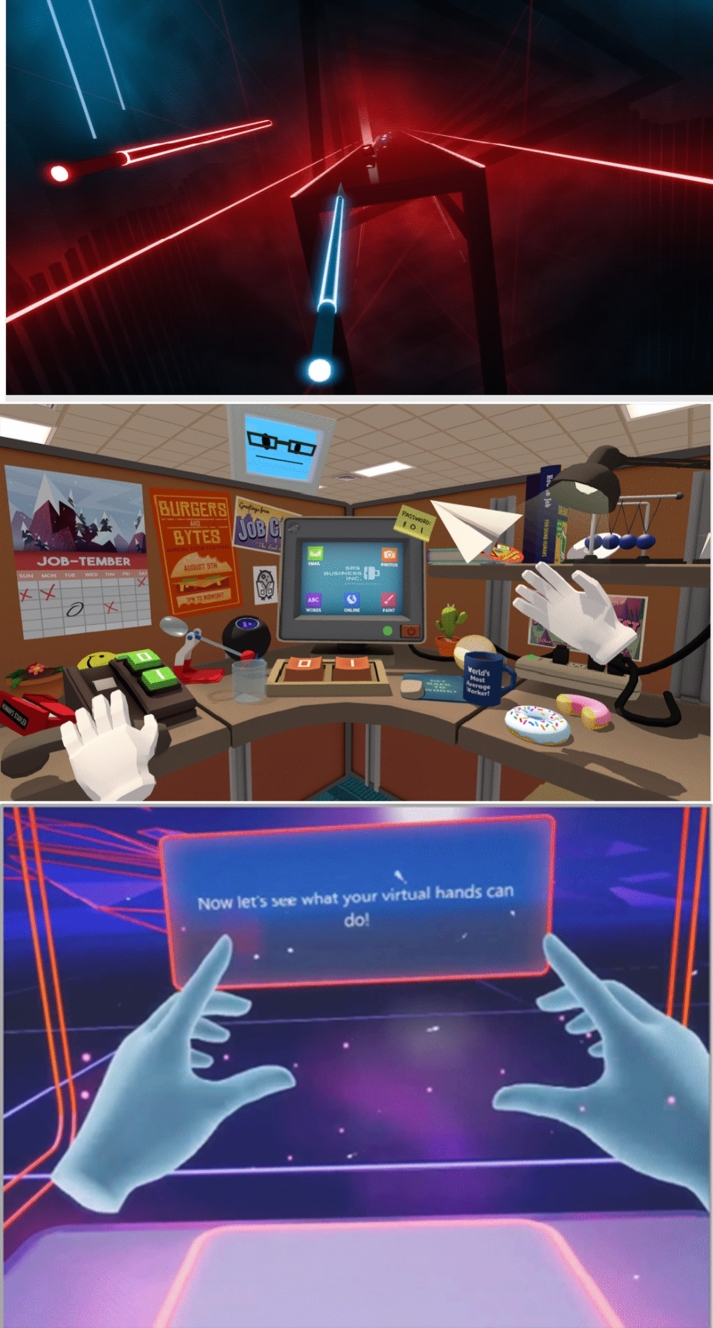
Images taken from each of the three games evaluated (Beat Saber, Job Simulator, and First Steps, from left to right), showing the image presented to users of the virtual hands. Images used by permission (Playstation 2018; Owlchemy Labs 2016a, b; Oculus 2019)

To evaluate the validity of the questionnaire, it is necessary to evaluate the reliability, validity, and other psychometric properties of the proposed questionnaire based on evidence from a large data set across multiple interactions. For this validation, we chose to use virtual reality games on the Oculus Quest platform. We selected the Oculus Quest and controllers (see Figs. 1 and 2) because questionnaire validation requires a large number of participants and the Quest is available as off-the-shelf hardware and has been adopted by a large number of users. Games were selected as the Quest is used primarily for that purpose and those applications provided us with the largest number of potential users. In addition to a large pool of potential participants, each virtual reality game selected provided a diverse but coherent experience in terms of manual interactions with the virtual world. We selected games based on their respective number of users (to ensure a reasonable sample size) and the ways in which hands were represented visually and used for interactions in these games. Based on these criteria, we selected three games from the Oculus Quest library. Beat Saber (Beat Games 2018) shows virtual swords that are controlled by the user; Job Simulator (Owlchemy Labs 2016a, b) represents hands as white gloves; and First Steps (Oculus 2019) represents hands in a manner that looks like human hands, although they are displayed in blue (see Fig. 2).

Beat Saber (Beat Games 2018) is a game where boxes (labeled with directional arrows) or walls fly toward users. The user’s goal is to use the swords held in their hands to either slice the box in the indicated direction (and in time to the beat of the music) or to duck out of the way of the approaching wall.

Job Simulator (Owlchemy Labs 2016a, b) is a game that allows users to simulate performing a job. We used the office worker scenario for this study, where users are asked, among other things, to use a computer mouse or correct sales figures in a report.

First Steps (Oculus 2019) is a tutorial designed to acquaint users with the Oculus controllers. There are opportunities to interact with cubes, ping pong balls, a tether ball, and to fly a blimp and a paper airplane.

### Study 1

#### Materials and apparatus

A final questionnaire with 16 items was created based on feedback from the cognitive interview study. Content validity of the questionnaire was examined and confirmed by subject matter experts and internal user study reviewers. The materials included the questionnaire, a 5-point Likert scale question regarding overall satisfaction with the game (“How was your overall experience the last time you played [specific game] on the Oculus Quest?”), and demographic questions. Versions of the questionnaire were then developed for each of the three games in order to tailor the questions to specific interactions in each of the games. Questions regarding these interactions were designed to be as comparable as possible across games. For example, a question focused on understanding the quality of the interaction was in the form of “How easy or difficult was it to [complete specific interaction] using [specific device]”. Further, two copies of each questionnaire were constructed with different response anchor directions. In one copy, the scale ranged from a “low user satisfaction” anchor (e.g., *very uncomfortable*) to a “high user satisfaction” anchor (e.g., *very comfortable*)”. In the other copy of the questionnaire, the anchor labels were reversed (e.g., from *very comfortable* to *very uncomfortable*.) All questions in a given questionnaire were anchored in the same direction. The final questionnaire can be seen in the “Appendix”.

Validation of the revised questionnaire was then conducted with a laboratory study and an online study using the same games but different procedures and participants. The Oculus Quest hardware was provided in the laboratory study.

#### Participants

For the online study, we reached out, by e-mail, to 7500 randomly selected Oculus Quest VR system users who agreed to be included in a database of users who might be approached for research studies. Those who were willing to participate in the survey were linked to the questionnaire. We received a total of 1284 responses, for an overall response rate of 16.7%; 10.7% completed the full survey. The final sample size was 799; 88% of the sample indicated they were male and 12% indicated they were female (other options presented were not selected by participants). The participants’ age ranges were reported as 18–24 (30%), 25–34 (28%), 35–44 (26%), 45 + (16%). When asked about their experience using VR, 94% indicated that they were actively using VR on a weekly basis. The total number of completed questionnaires for each game was 165, 362, and 211 for First Steps, Beat Saber, and Job Simulator, respectively.

For the laboratory study, individuals working within the company were solicited for participation. No compensation was provided to these participants. Twenty-one individuals agreed to serve in this study. The participants in this sample were 43% male and 57% female. Their ages were reported as 18–24 (29%), 25–34 (62%), and 35–44 (9%). In terms of VR experience, 38% reported that they were active on a weekly basis; 43% reported that they had played a game in VR less than five times total.

#### Procedure

Individuals who agreed to participate were provided with a consent form and asked whether they were 18 years or older. Individuals less than 18 years were thanked for their participation and exited from the survey. Those who indicated that they were older than 18 years of age were asked to indicate (from a list of possibilities) games they had played within the past week on the Oculus Quest. If they indicated that they had played any of First Steps, Beat Saber, or Job Simulator, they were asked to play one of those games. The game they were asked to play was selected by identifying the game which had the smallest population of users. Our goal was to get the largest sample size possible for each game. Participants played the game for 10 min, after which they were asked to complete the questionnaire associated with the selected game.

In the laboratory study, participants were first trained on the use of the controllers (where needed). After that, they played First Steps, Beat Saber and Job Simulator in a counter-balanced order. At the completion of a 10-min session for each game, they completed the questionnaire associated with that game.

#### Results

*Consistency across conditions* The data were first analyzed to ensure that the alternate labeling of the anchors (vs. from “high” anchor to “low” anchor) did not affect the data; this analysis confirmed no difference between the high- vs. low-anchor copies of the questionnaires, so data were combined across both versions.

Second, independent sample *t* tests were conducted to examine the pattern of responses across the laboratory and online studies. Average item responses were generally not significantly different between laboratory and online conditions; however, there were some exceptions. For Beat Saber, the reported feeling of being in control of the virtual hands was higher in the laboratory (4.7) than online (4.4), *t* (25) = − 3.35, *p* = 0.003. For Job Simulator, the evaluations of individual items were lower overall when assessed in the laboratory than online for 9 of the 16 items (using a *p* value of 0.05). It may be that the participants in the laboratory condition, who had a lower level of experience overall with VR, may have experienced more difficulty learning the interactions than the users in the online condition, leading to lower scores.

##### Factor structure

Factor analysis was used to identify which items on the questionnaire clustered together to form a factor and to determine the number of factors that best characterized the set of questions developed.

Factor Structure within Games: Each game’s questionnaire differed depending on the specific interactions users were asked to rate, which may have affected the ways in which the questions came together as factors. Thus, data for each game were analyzed separately. Scree plots using parallel analysis [7] were generated by game to examine the number of significant eigenvalues or latent factors in the online data. An examination of the scree plots suggested that the best solution for each game was a four-factor solution. An exploratory factor analysis assuming four factors was conducted separately for each game, using a principal axis with promax rotation. The variance explained by four factors were 0.40 (Beat Saber), 0.44 (First Steps), and 0.49 (Job Simulator). Following standard practice in conducting a factor analysis, the questions loading on a given factor were examined to determine the best label for each factor. Specifically, the items that loaded most heavily on a factor were examined to determine what label would best describe what they have in common, which may result in different levels of abstraction in the factor labels. For two of the games (Beat Saber and First Steps), the four factors were defined as.

- Quality of interaction​
- Assessment of task performance​
- Comfort
- Quality of the sensory enhancements

For Job Simulator, questions measuring the quality of sensory enhancements grouped with the first factor (quality of interaction), while items measuring object believability fell out as a separate factor. For the other two games, the items measuring object believability grouped with a different factor, as seen in Table 1.

**Table 1:** Labels associated with items in each factor for each game

Factor	Beat Saber	First steps	Job simulator
1	Quality of manual interaction & Object believability	Quality of manual interaction	Quality of manual interaction & Sensory enhancement
2	Assessment of Task Performance	Assessment of Task Performance & Object believability	Assessment of Task Performance
3	Comfort	Comfort	Comfort
4	Quality of sensory enhancement	Quality of sensory enhancement	Object believability

Factor Structure across Games: Our goal was to develop a questionnaire that could be tailored to interactions across several different input devices and forms of interactions. Thus, we wanted to determine whether the factors identified within each individual game would hold across the games. An exploratory factor analysis was conducted using the data from the online users across all games. We hoped that items that differed across games, but which were meant to measure the same construct, would hold together as a factor. The best solution using a principal axis with promax rotation approach was a five-factor solution:

- Quality of manual interaction​
- Assessment of task performance
- Quality of the sensory enhancements
- Object believability
- Comfort

Here, the first four factors address interaction-level user experience, while the last, Comfort, targets the holistic experience of the interaction. The variance explained was 0.46, with RMSEA = 0.038, which is considered a good model fit. This suggests that despite a focus on specific interactions for each game, the clusters of items hold consistently across all games. It also allowed the items measuring object believability to pull out as a separate factor. The final list of questions and response anchors are shown in “Appendix A”.

##### Instrument reliability

For all three games, reliability, as indicated by internal consistency of the instrument (Cronbach’s alpha), was in the range considered practically acceptable. Items in the final questionnaires were closely related to one another, with Cronbach’s alpha of 0.86 (Job Simulator), 0.80 (First Steps), and 0.78 (Beat Saber.)

We also examined whether removing any items might improve instrument reliability. The only question for which this was true was the cognitive load question. Removing the cognitive load question from the questionnaire increased alpha for two of the games (Beat Saber & Job Simulator) and had no impact on one (First Steps).

##### Sensitivity

We also were interested in the extent to which the questionnaire was sensitive to differences in the ease of user interaction. That is, the different games had very different representations of hands and different styles of interaction. Thus, the expectation was that there would be differences in the mean scores assigned to questions that targeted these aspects of the interactions. In fact, the mean user responses (on a 5-point Likert scale) varied by game for a number of questions. In all of the following comparisons (see Table 2), Tukey HSD was used to evaluate individual differences when the overall cross-game difference was significant (using *p* < 0.01 for all individual comparisons).

**Table 2:** Mean rating for each question on a 5-point Likert scale as a function of game (Beat Saber = BS, First Steps = FS, Job Simulator = JS). Difference column indicates significant differences (or lack thereof, “*ns*”) between pairs of means, using Tukey HSD

Question asked	Beat saber	First steps	Job simulator	Differences
Extent to which the virtual hands feel like my (real) hands?	3.88	3.79	3.68	(BS *ns* FS) > JS
How easy or difficult was it to interact with objects using the controllers?	4.54	4.51	4.21	(BS *ns* FS) > JS
How believable were the [x]?	4.36 [swords]	3.88 [paper airplane]	3.98 [computer mouse]	BS > (FS *ns* JS)
How consistent with interactions in the real world was interacting with [x]?	4.17 [swords]	3.87 [paper airplane]	3.63 [computer mouse]	BS > (FS *ns* JS)
How easy or difficult was it to learn how to [x]?	4.6 [slice boxes]	4.19 [throw the paper airplane]	4.16 [fix sales reports]	BS > (FS *ns* JS)
How easy or difficult was it to learn how to [x]?	4.56 [dodge walls]	3.96 [fly the blimp]	4.54 [make coffee]	(BS *ns* JS) > FS
How sure are you that you could successfully [x] again?	4.79 [slice boxes]	4.51 [fly the paper airplane]	4.50 [fix sales reports]	BS > (FS *ns* JS)

The virtual hands were reported to feel most like real hands in Beat Saber and least in Job Simulator, with a Tukey HSD showing no significant difference between Beat Saber and First Steps but a significant difference between ratings for both Beat and First Steps against Job Simulator. This was expected, given that the hands displayed in Job Simulator are more cartoonish than the hands shown in First Steps (as shown in Fig. 2) and they require different gestures than real hands. These speculations were supported by the qualitative explanations provided by participants (and shown in Tables 3 and 4). The top response for why the virtual hands did not feel like participants’ own hands was that the controllers required a different form of gesture than in real-world interactions with the specified device; the next most common response was the look of the virtual hands.

**Table 3:** The percentage of participants who selected each of the three top reasons why participants felt that the virtual hands felt like their real hands, as a function of game being played

Reason: game	Intuitive control	Fast response	Same gestures
Job Simulator	58	74	61
First Steps	65	68	65
Beat Saber	60	71	70

**Table 4:** The percentage of participants who selected each of the three top reasons that the virtual hands did not feel like their real hands, as a function of game being played

Reason: game	Different gestures	Did not look like my hands	Poor haptics
Job simulator	46	29	0
First steps	47	23	0
Beat saber	30	0	22

Interestingly, no hands are shown for Beat Saber; however, the controllers are held in the hand in the same manner as one would hold a real sword, which may account for the high rating here. This was reflected in the frequent mention of consistency of the gestures as an explanation for both positive and negative evaluations (see Tables 3 and 4).

To the question of how easy or difficult it was to interact using the controllers, participants found them equally easy/difficult to user for Beat Saber and First Steps, and both were reported to be easier to use than in Job Simulator. The degree to which the gestures required to interact with the objects are similar to, or different from, actual hand gestures seems to be driving the cross-game discrepancies, as the gestures used in Job Simulator are often quite different from those used in the real world (e.g., “typing” on the keyboard). In addition, the believability of objects, or interactions with objects, differed across games. The swords in Beat Saber were rated as more believable than the computer mouse in Job Simulator or the paper airplanes in First Steps.

Interacting with the swords in Beat Saber was rated as more consistent with real-world experiences than​ interacting with either the computer mouse in Job Simulator or the paper airplanes in First Steps. These findings were in line with our expectations given that interactions with the swords by holding onto the controllers were more consistent with the actions needed to interact with them in the real world.

The reported ability to learn how to interact with objects also differed across games, in ways that made sense. Slicing boxes was thought to be easier to learn than either throwing the paper airplane or fixing sales reports on the computer. Dodging walls and making coffee were considered easier to learn than flying the blimp​​. When asked how sure they were that they could be successful in doing a task again, participants reported it would be easier to slice boxes than to either fly the paper airplane or fix sales reports.

##### Relationship to overall satisfaction

Although our focus was on specific interactions, we hoped that responses would also be related to overall satisfaction. The extent to which the facets of user experience measured in the questionnaire was related to overall user experience was examined in the correlations between questionnaire items and the question about overall satisfaction.

For Beat Saber​, object believability *(r* = 0.40)​ was the most highly correlated factor with overall satisfaction. For First Steps​, system responsiveness (*r* = 0.37)​, limb ownership (r = 0.32)​, and object believability *(r* = 0.44)​ were all related to overall satisfaction. For Job Simulator, system responsiveness (*r* = 0.41) and sounds that enhance the experience (*r* = 0.4) were most highly related to overall satisfaction. All these correlations were statistically significant after controlling for a family-wise type I error rate of 0.05.

When we look at the factors that are least related to overall satisfaction, we see that for all three games, cognitive load was least related to satisfaction (Beat Saber = 0.01; First Steps = 0.15; Job Simulator = 0.02). This may well be due to the lack of internal consistency found with this item. In addition, learnability was only weakly related to satisfaction for Beat Saber (*r* = 0.07); learnability was only weakly related to satisfaction (*r* = 0.1) for Job Simulator. These correlations were not statistically significant.

### Study 2

After completing the first study, we recognized that we were not measuring some constructs that we felt were important to assess. Specifically, in assessing task performance, we felt that ease of learning and a self-assessment of one’s ability to play the game were missing. We also wanted to validate our factor structure using a new sample. Thus, we conducted a second study following a similar procedure to that used in the first. Given restrictions on data collection imposed by COVID-19, we replicated only the online portion of the first study.

#### Materials and apparatus

A 21-item questionnaire was developed for this study. The questionnaire included the 16 items from the questionnaire used in Study 1 and 5 new items.

- When [slicing through boxes], did the controllers worked the way you expected?
- I could achieve what I wanted to while playing this game.
- I felt capable when playing the game.
- How easy or difficult was it to learn to operate the Quest?
- I needed to learn a lot of things before I could start playing the game.

#### Procedure

The experimental procedure replicated the online portion of Study 1.

#### Participants

For this study, 15,000 participants who have experience with the Oculus Quest VR system were solicited via email. This number solicited was higher than in the previous study due to the low response rate in the initial study and a desire to ensure a sufficient number of responses for psychometric analyses. Those who were willing to participate in the survey were linked to the questionnaire. We received a total of 1581 responses, for an overall response rate of 10.5%; 3.8% completed the full survey. The final sample size was 571; 80% of the sample indicated they were male and 17% indicated they were female (other options were selected by 3%). The participants’ age ranges were reported as 18–24 (32%), 25–34 (20%), 35–44 (25%), 45 + (23%). When asked about their experience using VR, 95% indicated that they were active using VR on a weekly basis. The total number of completed questionnaires for each game was 134, 250, and 187 for First Steps, Beat Saber, and Job Simulator, respectively.

#### Results

To enable the following factor analysis, we converted the 5- and 9-point scales used in the items to a common 7-point scale for ease of comparison across items. To do this, we used the following formula (IBM Support 2020): 1 + (original score − 1) * (7 − 1)/(original scale points − 1). All of the descriptive statistics (e.g., mean) reported in the sections below were calculated based on converted values.

##### Factor structure

Following the same analysis style as in Study 1, data from the three games were analyzed separately. An examination of scree plots using Horn’s parallel analysis suggested that the best solution for each game was a five-factor solution. An exploratory factor analysis assuming five factors was conducted separately for each game, using a principal axis with promax rotation. The variance explained by five factors was 0.39 (Beat Saber), 0.55 (First Steps), and 0.48 (Job Simulator). The model fit indicator RMSEA assuming a five-factor structure ranged from 0.045 (Beat Saber), 0.053 (First Steps), to 0.056 (Job Simulator). All these RMSEA values indicated good model fit.

Despite the imbalance of sample sizes across the three games, the three factor structures that emerged from the datasets were largely consistent. Again, the questions loading on each factor were examined to create a label for that factor. The first four factors address interaction-level user experience, while the last, Comfort, targets the holistic experience of the interaction.

The five factors based on the Beat Saber dataset were defined as.

- Quality of manual interaction
- Assessment of task performance
- Quality of the sensory enhancements
- Consistency with expectations (previously “object believability” in Study 1)
- Comfort

The factor structure of First Steps differed slightly from that of Beat Saber. Two questions that loaded on the consistency with expectations factor and three questions loading on the assessment of task performance factor loaded on a different factor. For Job simulator, two questions loading on the consistency with expectations factor and two questions loading on the assessment of task performance factor loaded on factors that are different from the structure in Beat Saber.

Despite these small differences, the results suggest that the clusters of items held together well across all three games. The factor structure is also largely consistent with results of Study 1. The only inconsistency in the clustering was found in two questions loading on the assessment of task performance factor, including “How easy or difficult was it to swing the swords using the controllers” and “How much mental effort did you invest in the task of slicing through a box”. In addition, the new item “I needed to learn a lot of things before I could start playing the game” was not clearly associated with any factor based on its low factor loading values.

The questions, excluding the three questions with inconsistent or low factor loading and the question on overall satisfaction, are shown in “Appendix C” along with their response anchors and associated factor groups.

##### Instrument reliability

We found evidence of reasonable internal reliability of the questionnaire, with slightly stronger values for Cronbach’s alpha in this study (Job Simulator (0.86), First Steps (0.88), and Beat Saber (0.79)) than in Study 1.

We found that removing two items would improve internal reliability: “I needed to learn a lot of things before I could start playing the game;” and, consistent with Study 1, “How much mental effort did you invest in the task of slicing through a box”.

##### Sensitivity

Again, it was important to ensure that scores assigned to specific interactions reflected perceived differences across the different forms of interaction. Here, the mean user responses to individual items varied by game for 10 of 21 questions using Tukey HSD for pairwise comparison. Table 5 presents the mean response scores for each of those 10 questions, with higher scores indicating a more positive experience.

**Table 5:** Mean rating for each question on a 7-point Likert scale as a function of game (Beat Saber *ns* BS, First Steps *ns* FS, Job Simulator *ns* JS). Difference column indicates significant differences (or lack thereof, “*ns*”) between pairs of means, using Tukey HSD

Question Asked	Beat Saber (BS)	First Steps (FS)	Job Simulator (JS)	Sig. Diff.
Were your interactions with the [x] consistent or inconsistent with your real-world experiences?	5.57 [swords]	5.19	5.09	BS > JS
Did you find the [x] believable?	5.28 [swords]	4.97	4.40	BS *ns* FS > JS
When [x], did the controllers worked the way you expected?	5.81 [slicing through boxes]	5.39	5.68	BS > FS
How easy or difficult was it to learn how to [x]?	6.22 [avoid the oncoming walls]	5.35	6.04	BS *ns* JS > FS
How sure are you that you could successfully [x] if asked to do it again?	6.42 [slice through boxes in indicated direction]	6.09	6.07	BS > JS
I needed to learn a lot of things before I could start playing the game	4.64	4.00	4.45	BS *ns* JS > FS
How much mental effort did you invest in the task of [x]?	4.55 [slicing through a box]	5.15	5.01	FS *ns* JS > BS
How comfortable or uncomfortable was the weight of the headset?	4.13	4.14	4.59	JS > BS
Did the sounds that happened when you interacted with virtual objects (for example, when you [x]) enhance or detract from your enjoyment of the application?	6.17 [sliced through a box]	6.54	5.99	FS > BS *ns* JS
Did the feelings of touch when you interacted with virtual objects enhance or detract from your enjoyment of the application?	5.98	6.07	5.65	BS *ns* FS > JS

Interacting with the swords in Beat Saber was rated as more consistent with real-world experiences than interacting with either the computer mouse in Job Simulator or the paper airplane in First Steps. These findings were in line with expectations given that the controllers are held in the same way that swords might be held in the real world.

When asked about the quality of the sensory experience, participants in Beat Saber and First Steps reported significantly higher values than those in Job Simulator that feelings of touch enhanced their experience.

##### Relationship to overall satisfaction

To understand whether ratings for individual items are related to the overall user experience, we examined the correlations between questionnaire items and the question about overall satisfaction. Items from the factor “consistency with expectations” were highly correlated with overall satisfaction across all three games.

For Beat Saber, items on the consistency with expectations factor were the most highly correlated factor with overall satisfaction, with *r* = 0.38 for both “I could achieve what I wanted to while playing this game” and “I felt capable when playing the game”. For First Steps, sensory enhancement with sound (*r* = 0.51), consistency with expectations (“I could achieve what I wanted to while playing this game, *r* = 0.48), and quality of interaction (“To what extent did you feel that the virtual hands were in the location of your (real) hands?”, *r* = 0.47) were all related to overall satisfaction. For Job Simulator, consistency with expectations (“I felt capable when playing the game”, *r* = 0.41; “I could achieve what I wanted to while playing this game”, *r* = 0.40), assessment of task performance (“How easy or difficult was it to interact with virtual objects in the game using the controllers”, *r* = 0.43), and sensory enhancement with sound (*r* = 0.41) were most highly related to overall satisfaction. All these correlations were statistically significant after controlling for family-wise type I error of 0.05.

When we look at the factors that are least related to overall satisfaction, we see that for all three games, cognitive load was least related to satisfaction (Beat Saber = 0.04; First Steps = 0.03; Job Simulator = 0.04). This may well be due to the lack of internal consistency found with this item. In addition, “I needed to learn a lot of things before I could start playing the game” in the assessment of task performance factor also showed low correlation across all games (Beat Saber = 0.02; First Steps = − 0.11; Job Simulator = − 0.09). These correlations were not statistically significant.

## Pilot testing of the questionnaire with AR users

Although the validation studies provided substantial evidence for the statistical validation of the questionnaire, we recognize that evidence is limited in its scope of generalizability because the experiences (three games) took place in a virtual environment. We wanted to examine the feasibility of using the questionnaire in an augmented environment. We thought this would be possible because AR platforms are similar to VR in that they enable users to interact with virtual elements projected on a head-mounted display screen (glasses/headset). However, the user experience in AR may be different from that in VR because users can see the real world in addition to the VR world. We conducted a small study following a similar procedure to the online portion of the first two studies.

Unfortunately, due to the small number of AR users and COVID restrictions in bringing people into the laboratory, it was not possible to collect sufficient data to perform a factor analysis on the questionnaire data. Instead, the data were compared to those collected in the previous studies.

### Materials and apparatus

We adapted the 21-item questionnaire from Study 2 based on the tracking mechanism of AR platforms. We also substituted wordings in the items to align with the application that we asked participants to use in this study. The following items from Study 2 were revised.

- *Original:* How easy or difficult was it to swing the swords using the controllers?
  *Revision:* How easy or difficult was it to interact with virtual objects in the application using your hand gestures?
- *Original:* To what extent did the virtual hands feel like your hands?
  *Revision:* To what extent did it feel natural using your hands to interact with virtual objects?
- *Original:* To what extent did you feel that you were in control of what the virtual hands did?
  *Revision:* To what extent did you feel that you were in control of your gaze in selecting target items on the control panel?
- *Original:* To what extent did you feel that the virtual hands were in the location of your (real) hands?
  *Revision:* To what extent did you feel that you were in control of your gaze in selecting letters on the keyboard?
- *Original:* Did you find the swords believable?
  *Revision:* Did you find the process of rotating the text box believable?

### Procedure

We reached out by e-mail and forum posts to recruit individuals who own a Hololens 1 or Hololens 2. If a user agreed to participate, they were provided with a consent form and asked whether they were 18 years or older. Individuals less than 18 years old were thanked for their participation and exited from the survey. Those who indicated that they were older than 18 years of age were asked to install the application “Type in Space” (a free app) from the Microsoft store on their AR device.

Participants were instructed to explore the application by completing a series of tasks. They were asked to pick three objects in their room, name the objects in the text boxes, and manipulate the text boxes (change text color, change text size, and rotate text box). After finishing these actions, participants evaluated their experience in the 21-item questionnaire. Once they completed the questionnaire, they were thanked and debriefed.

### Participants

We received a total of 31 responses from Hololens users, and all completed the questionnaire. Among the 31 users, 77% of the sample indicated they were male and 23% indicated they were female (no participants selected other options presented). The participants’ age ranges were reported as 18–24 (35%), 25–34 (13%), 35–44 (26%), 45 + (26%). In terms of experience in AR, 55% indicated that they use AR on a weekly basis.

### Results

Due to the small sample size, we could not validate the psychometric properties of the questionnaire in the context of AR interactions. However, we were able to compare the responses to the same items between the AR and VR interactions tested to gauge the extent to which user experience differs in AR and VR technologies. We compared responses from VR users who played Beat Saber in Study 2 with Hololens users in this study (see Table 6).

**Table 6:** Mean rating for each question as a function of VR (Beat Saber) versus AR (Hololens). Difference column indicates p value associated with the difference between pairs of means

	VR	AR	Sig. Diff.
*Consistency with expectations*			
Were your interactions with the swords consistent or inconsistent with your real-world experiences?	5.57	4.97	*p* = .07
Did you find the swords believable?	5.28	4.53	*p* = .03
When slicing through boxes, did the controllers worked the way you expected?	5.81	5.26	*p* = .09
I could achieve what I wanted to while playing this game	6.13	5.26	*p* = .01
I felt capable when playing the game	6.44	5.29	***p* = .001**
*Assessment of Task Performance*			
How easy or difficult was it to learn how to slice through a box in the indicated direction?	6.04	5.16	*p* = .01
How easy or difficult was it to learn how to avoid the oncoming walls?	6.22	5.06	***p* < .001**
How sure are you that you could successfully slice through boxes in the indicated direction if asked to do it again?	6.42	5.55	*p* = .03
How easy or difficult was it to learn to operate the Quest?	6.10	4.84	***p* < .001**
I needed to learn a lot of things before I could start playing the game	4.64	3.65	*p* = .01
How easy or difficult was it to swing the swords using the controllers?	5.92	3.87	***p* < .001**
How much mental effort did you invest in the task of slicing through a box?	4.55	4.63	*p* = .79
*Comfort*			
How comfortable or uncomfortable was the weight of the headset?	4.13	4.84	*p* = .05
How comfortable or uncomfortable was the fit of the headset?	4.43	5.13	*p* = .04
*Quality of hand interactions*			
To what extent did you feel that you were in control of what the virtual hands did?	4.92	4.43	*p* = .23
To what extent did you feel that the virtual hands were in the location of your (real) hands?	5.44	4.00	*p* = .004
How fast or slow did the virtual world respond to your actions?	5.65	5.13	*p* = .12
Were the virtual world's responses to your actions consistent or inconsistent with your real-world experiences?	5.61	4.84	*p* = .03
To what extent did the virtual hands feel like your hands?	4.28	4.10	*p* = .52
*Quality of sensory enhancements*			
Did the sounds that happened when you interacted with virtual objects (for example, when you sliced through a box) enhance or detract from your enjoyment of the application?	6.17	5.74	*p* = .05
Did the feelings of touch when you interacted with virtual objects enhance or detract from your enjoyment of the application?	5.98	5.22	*p* = .01

On average, ratings from Hololens users were lower than those from VR users (see Table 6 below). However, the differences were not statistically significant for the majority of questionnaire items after controlling for familywise Type I error rate. The only four items showing a significant difference (with boldface p values in Table 6) were asking about users’ assessment of task performance and whether they felt capable when playing the game.

We did observe two trends about user experience across these two types of technologies. First, the experience in VR seems to be better than that in AR based on users’ evaluations of consistency with expectations, assessment of task performance, quality of interaction, and quality of sensory enhancement. However, the AR technology we used received a better comfort rating than the technology we used for the VR experience, which may be due to the differences in hardware design between the Hololens and Oculus Quest.

## General discussion

As new forms of interactions are developed for use in AR/VR, it is important to be able to assess the quality of the interactions methodically. This paper describes the development of a questionnaire, the Customized Interactions Questionnaire, CIQ (shown in “Appendix C”) designed to measure subjective reactions of users to specific modes of interacting with objects as found in VR and AR games and which can be customized to new interaction methods.

The factors we identified through this process are important in understanding how useful devices are for completing specific tasks from multiple viewpoints: quality of interactions, subjective assessment of ability to perform the specified task using the specified mode of interaction, comfort in performing the tasks, quality of the sensory enhancements (sound, touch) provided by the mode of interaction, and the consistency of the interaction with user expectations.

We started with a cognitive interview study to develop the strongest question stems and response alternatives for the questionnaire being developed. We then turned to psychometric evaluations to evaluate the questionnaires. Specifically, we were interested in the internal consistency of the questionnaire, the factor structure present in the questionnaire, and the sensitivity of the questionnaire, particularly as it related to understanding the quality of the specific interactions users had with objects in the games and how easily users perceived they could accomplish tasks using that interaction method.

Most importantly, items in the questionnaire were sensitive to differences across games in the mode of interacting with objects. Thus, we believe that this questionnaire will be useful in evaluating new interactions and prototypes for interaction as they are developed.

The analyses further suggested that not all questions loaded as strongly on the identified dimensions as some others did. The appendix identifies two questions per factor that loaded most strongly and that could be used to develop a smaller set of questions for assessing these five factors.

## Limitations

There are several caveats to our conclusions. First, the imbalance in the number of responses across games in the VR studies may have introduced biases into the statistical analyses. For example, there were more responses to the Beat Saber version of the questionnaire than the other two games in both studies.

Second, the questionnaire design used in the VR studies highlighted manual interaction with objects using a controller as the primary input method, leaving a question about the ability of the questionnaire to be used for other, new forms of interactions with objects. The AR study addressed this to some extent, allowing users to interact with their hands (no controller) and using eye gaze as a selection method. The questionnaire was adapted for these uses and the data suggested that the instrument were sensitive to differences in performance. However, the evaluation of this AR-adapted questionnaire used a limited number of respondents and our conclusions here should be considered preliminary and inconclusive.

Future studies (post-pandemic) should make it easier for us to evaluate the questionnaire through in-laboratory user studies and will provide a better opportunity to gather a sufficient sample for evaluating the AR-adapted questionnaire. They should also allow us the ability to examine other forms of system input, such as voice commands.

## Conclusion

Overall, our results suggest we were able to develop an instrument that can be customized for specific input devices and that is useful in evaluating the quality of interactions with objects in AR/VR spaces. The five-factor solution identified not only meets the theoretical requirements of a reliable and valid questionnaire, but also ensures a consistent interpretation of the questions.

The questionnaire also allows researchers to evaluate other features relevant to user satisfaction, including how consistent the interactions were with their expectations (believability), quality of the sensory enhancements present in the environment, and comfort of these interactions. We also believe that the questionnaire can be modified to fit new scenarios and interaction technologies by filling in the appropriate blanks within each question stem (e.g., “how easy or difficult was it to [complete specific action] using [specific technology]?”). When we combined questions across games, the specific questions targeted at measuring the quality of manual interaction continued to form a cohesive factor.

The consistency of the data across studies supports the notion that this questionnaire could also be extended to support interactions beyond those we focused on, which was interacting with objects. This extended CIQ, or E-CIQ, (which has not been validated) is shown in “Appendix D” and could be used to evaluate interactions beyond those when a user engages in a virtual or augmented environment using something other than their hands. This version of the questionnaire suggests how items such as “hand” could be replaced with the appropriate body/device part. For example, the question “To what extent did you feel that the virtual hands were in the location of your (real) hands” could be adapted to “To what extent did you feel that the virtual body was in the location of your (real) body” to assess body-centered experiences/interactions. Another example, which was used in our AR experiment, substituted “To what extent did you feel that you were in control of your gaze in selecting target items on the control panel?” for the original question of “To what extent did you feel that you were in control of what the virtual hands did”. The validation of this instrument remains for future research.

## Data Availability

Not available.

## Appendix A

Questions from the survey, showing the factor grouping of questions, and the anchors associated with the question. Where questions were tailored to a specific game, the unique language inserted is shown in square brackets for each game [BS = Beat Saber, FS = First Steps, JS = Job Simulator]. Items in bold loaded most heavily on that factor. The item designed to measure cognitive load (shown in italics) will be removed from future versions of the instrument as it demonstrated low internal consistency and the lowest correlation of all items with overall satisfaction (Table 7).

**Table 7:** Survey used in study 1

Factor	Question	Anchors
Quality of Interactions	Were the virtual world’s responses to your actions consistent or inconsistent with your expectations?	Very inconsistent—Very consistent
Quality of Interactions	To what extent did you feel that the virtual hands were in the location of your (real) hands?	Not at all—Very much
Quality of Interactions	How fast or slow did the virtual world respond to your actions?	Very slow—Very fast
Quality of Interactions	How easy or difficult was it to interact with virtual objects in the game using [insert] [controllers, FS, JS]	Very difficult—Very easy
Quality of Interactions	To what extent did the virtual hands feel like your hands?	Not at all like my hands—Just like my hands
Quality of Interactions	To what extent did you feel that you were in control of what the virtual hands did?	Not at all—Very much
Comfort	How comfortable or uncomfortable was the fit of the headset?	Very uncomfortable—Very comfortable
Comfort	How comfortable or uncomfortable was the weight of the headset?	Very uncomfortable—Very comfortable
Assessment of Task Performance	*How much mental effort did you invest in the task of [insert]? [slicing through a box, BS] [throwing a paper airplane, FS] [fixing sales report numbers, JS]*	Very high—Very low
Assessment of Task Performance	How easy or difficult was it to learn how to [insert]? [slice through a box in the indicated direction, BS] [throw a paper airplane, FS] [fix sales report numbers, JS]	Very difficult—Very easy
Assessment of Task Performance	How easy or difficult was it to learn to [insert]? [to avoid the oncoming walls, BS] [how to fly the blimp, FS] [how to make coffee, JS]	Very difficult—Very easy
Assessment of Task Performance	How sure or unsure are you that you could successfully [insert] if asked to do it again? [slice through boxes in the indicated direction, BS] [throw a paper airplane, FS] [fix sales report numbers, JS]	Very unsure—Very sure
Object Believability	Were your interactions with the [insert] consistent or inconsistent with your real-world experiences? [swords, BS] [paper airplanes, FS] [computer mouse, JS]	Very inconsistent—Very consistent
Object Believability	Did you find the [insert] believable? [swords, BS] [paper airplanes, FS] [computer mouse, JS]	Not at all believable—Very Believable
Quality of the Sensory Enhancements	Did the sounds that happened when you interacted with the virtual objects (for example, [insert]) enhance or detract from your enjoyment of the application? [when you sliced through a box, BS] [when you dropped a cube on the desk, FS] [when you eat food, JS]	Greatly detracted—Greatly enhanced
Quality of the Sensory Enhancements	Did the feelings of touch when you interacted with virtual objects enhance or detract from your enjoyment of the application?	Greatly detracted—Greatly enhanced

## Appendix B

Questions from pilot testing with AR users, showing question stems and anchors (Table 8).

**Table 8:** Survey used in pilot testing with AR users

Question	Anchors
Were the virtual world’s responses to your actions consistent or inconsistent with your real-world experience?	Very inconsistent—Very consistent
To what extent did you feel natural using your hands to interact with virtual objects?	Not natural at all—Extremely natural
How fast or slow did the virtual world respond to your actions?	Very slow—Very fast
How easy or difficult was it to interact with virtual objects in the application using your hand gestures?	Very difficult—Very easy
To what extent did the virtual hands feel like your hands?	Not at all like my hands—Just like my hands
To what extent did you feel that you were in control of your gaze in selecting target items on the control panel?	Not in control at all—Extremely in control; Does not apply because I use Hololens 2
To what extent did you feel that you were in control of your gaze in selecting letters on the keyboard?	Not in control at all—Extremely in control; Does not apply because I use Hololens 2
How comfortable or uncomfortable was the fit of the headset?	Very uncomfortable—Very comfortable
How comfortable or uncomfortable was the weight of the headset?	Very uncomfortable—Very comfortable
How much mental effort did you invest in the task of placing a text box at your intended location?	Very very high—Very very low
How easy or difficult was it to learn how to place a text box at your intended location?	Very difficult—Very easy
How easy or difficult was it to learn how to rotate a text box?	Very difficult—Very easy
How sure are you that you could successfully place a text box at your intended location if asked to do it again?	Very unsure—Very sure
Were your interactions with the text box consistent or inconsistent with your real-world experiences?	Very inconsistent—Very consistent
Did you find the process of rotating the text box believable?	Not at all believable—Very Believable
Did the sounds that happened when you interacted with virtual objects (for example, when you selected a key on the keyboard)) enhance or detract from your enjoyment of the application?	Greatly detracted—Greatly enhanced
Did the feelings of touch when you interacted with virtual objects enhance or detract from your enjoyment of the application?	Greatly detracted—Greatly enhanced
How easy or difficult was it to learn to operate the Hololens?	Very difficult—Very easy
I needed to learn a lot of things before I could start using this application	Strongly disagree—Strongly agree
I felt capable when using this application	Strongly disagree—Strongly agree
I could achieve what I wanted to while using this application	Strongly disagree—Strongly agree
When placing a text box at your intended location, did the interaction work the way you expected?	Not at all—Extremely
How was your overall experience of using Type in Space on Hololens this time?	Very negative—Very positive

## Appendix C

Fully validated version of the CIQ with 17 questions based on the results of Study 2, showing the revised factor grouping of questions and the anchors. Where questions were tailored to a specific game, some examples of items that could be used to fill those items are shown, drawn from those we used in the validation (Table 9).

**Table 9:** Customizable interaction questionnaire (CIQ)

Factor	Question	Anchors
Quality of Interactions	Were the virtual world’s responses to your actions consistent or inconsistent with your expectations?	Very inconsistent—Very consistent
Quality of Interactions	To what extent did you feel that the virtual hands were in the location of your (real) hands?	Not at all—Very much
Quality of Interactions	How fast or slow did the virtual world respond to your actions?	Very slow—Very fast
Quality of Interactions	To what extent did the virtual hands feel like your hands?	Not at all like my hands—Just like my hands
Quality of Interactions	To what extent did you feel that you were in control of what the virtual hands did?	Not at all—Very much
Comfort	How comfortable or uncomfortable was the fit of the headset?	Very uncomfortable—Very comfortable
Comfort	How comfortable or uncomfortable was the weight of the headset?	Very uncomfortable—Very comfortable
Assessment of Task Performance	How easy or difficult was it to learn how to [insert]? [e.g., slice through a box in the indicated direction, throw a paper airplane, fix sales report numbers]	Very difficult—Very easy
Assessment of Task Performance	How easy or difficult was it to learn to operate the [insert]? [e.g., Quest]	Very difficult—Very easy
Assessment of Task Performance	How sure or unsure are you that you could successfully [insert] if asked to do it again? [e.g., slice through boxes in the indicated direction, throw a paper airplane, fix sales report numbers]	Very unsure—Very sure
Consistency with Expectation	Were your interactions with the [insert] consistent or inconsistent with your real-world experiences? [e.g., swords, paper airplanes, computer mouse]	Very inconsistent—Very consistent
Consistency with Expectation	Did you find the [insert] believable? [e.g., swords, paper airplanes, computer mouse]	Not at all believable—Very Believable
Consistency with Expectation	I felt capable when playing the game	Strongly disagree—Strongly agree
Consistency with Expectation	I could achieve what I wanted to while playing this game	Strongly disagree—Strongly agree
Consistency with Expectation	When [insert], did the controllers worked the way you expected? [e.g., slicing through boxes, throwing a paper airplane, fixing sales report numbers]	Not at all—Extremely
Quality of the Sensory Enhancements	Did the sounds that happened when you interacted with the virtual objects (for example, [insert]) enhance or detract from your enjoyment of the application? [e.g., when you sliced through a box, when you dropped a cube on the desk, when you eat food]	Greatly detracted—Greatly enhanced
Quality of the Sensory Enhancements	Did the feelings of touch when you interacted with virtual objects enhance or detract from your enjoyment of the application?	Greatly detracted—Greatly enhanced

## Appendix D

Extended Customizable version of CIQ allowing for other forms of interactions in AR/VR (Table 10).

**Table 10:** Extended customizable interaction questionnaire (E-Ciq)

Factor	Question	Anchors
Quality of Interactions	Were the virtual world’s responses to your actions consistent or inconsistent with your expectations?	Very inconsistent—Very consistent
Quality of Interactions	To what extent did you feel that the virtual [body part] was in the location of your real [body part]? [e.g., hands, head]	Not at all—Very much
Quality of Interactions	How fast or slow did the virtual world respond to your actions?	Very slow—Very fast
Quality of Interactions	To what extent did the virtual [body part] feel like your [body part]? [e.g., hands, head]	Not at all like my hands—Just like my hands
Quality of Interactions	To what extent did you feel that you were in control of what the virtual [body part] did? [e.g., hands, head]	Not at all—Very much
Comfort	How comfortable or uncomfortable was the fit of the [device]? [e.g., headset, glove]	Very uncomfortable—Very comfortable
Comfort	How comfortable or uncomfortable was the weight of the [device]? [e.g., headset, glove]	Very uncomfortable—Very comfortable
Assessment of Task Performance	How easy or difficult was it to learn how to [do x]? [e.g., slice through a box in the indicated direction, fix sales report numbers]	Very difficult— Very easy
Assessment of Task Performance	How easy or difficult was it to learn to operate the [insert]? [e.g., Quest]	Very difficult—Very easy
Assessment of Task Performance	How sure or unsure are you that you could successfully [do x] if asked to do it again? [e.g., slice through boxes in the indicated direction, throw a paper airplane]	Very unsure—Very sure
Consistency with Expectation	Were your interactions with the [insert] consistent or inconsistent with your real-world experiences? [e.g., swords, computer mouse]	Very inconsistent—Very consistent
Consistency with Expectation	Did you find the [insert] believable? [e.g., swords, paper airplanes, computer mouse]	Not at all believable—Very Believable
Consistency with Expectation	I felt capable when playing the game	Strongly disagree—Strongly agree
Consistency with Expectation	I could achieve what I wanted to while playing this game	Strongly disagree—Strongly agree
Consistency with Expectation	When [doing x], did the [interaction device] work the way you expected? [doing x, e.g., slicing through boxes, throwing an paper airplane] [interaction device, e.g., controllers, mouse, head cursor]	Not at all—Extremely
Quality of the Sensory Enhancements	Did the [sensory feedback] that happened when you interacted with the virtual objects (for example, [insert]) enhance or detract from your enjoyment of the application? [e.g., did the vibrations…when you sliced through a box; did the sounds…when you dropped a cube on the desk]	Greatly detracted—Greatly enhanced
Quality of the Sensory Enhancements	Did the feelings of [sensory feedback] when you interacted with virtual objects enhance or detract from your enjoyment of the application? [e.g., touch]	Greatly detracted—Greatly enhanced

## References

[CR6] Beat Games (2018) Beat Saber. Retrieved 19 Aug 2019 from https://www.oculus.com/experiences/rift/1304877726278670/?locale=en_US

[CR1] Brooke J, Thomas B, Weerdmeester BA, McClelland IL, Jordan PW (1996). SUS: a quick and dirty usability scale. Usability evaluation in industry.

[CR2] Cai T (2016) Perceiving motion patterns. In: Instinctive computing. Springer, London

[CR3] Chin JP, Diehl VA, Norman KL (1987) Development of an instrument measuring user satisfaction of the human-computer interface. In: Proceedings of ACM CHI '88, Washington, DC, pp 213–218

[CR4] Dickie C, Hart J, Vertegaal R, Eiser A (2006) LookPoint: an evaluation of eye input for hands-free switching of input devices between multiple computers. In: OZCHI 2006 proceedings. Sydney, Australia

[CR5] Fahmi F, Tanjung K, Nainggolan F, Siregar B, Mubarakah N, Zarlis M (2020) Comparison study of user experience between virtual reality controllers, leap motion controllers, and senso glove for anatomy learning systems in a virtual reality environment. In: 2020 IOP conference series: materials science and engineering, pp 851. doi:10.1088/1757-899X/851/1/012024.

[CR7] Haptx (2019) Retrieved September 2, 2019 from: https://haptx.com/

[CR8] Hart SG, Staveland LE (1988) Development of NASA-TLX (Task load index): results of empirical and theoretical research. In: Hancock PA, Meshkati N (eds) Human mental workload, North-Holland, pp 139–183. doi:10.1016/S0166-4115(08)62386-9.

[CR9] Horn JL (1965). A rationale and test for the number of factors in factor analysis. Psychometrika.

[CR10] IBM Support (2020) Transforming different Likert scales to a common scale. https://www.ibm.com/support/pages/transforming-different-likert-scales-common-scale. Accessed 4 Aug 2021.

[CR11] ISO 9241–410 (2008) Ergonomics of human-system interaction—part 410: design criteria for physical input devices

[CR12] ISO/TS 9241–411 (2012) Ergonomics of human-system interaction—part 411: evaluation methods for the design of physical input devices

[CR13] Karev K (2018) Technology: part 7—controllers and haptics. Medium. https://medium.com/@spammaleros/behold-the-next-generation-vr-technology-part-7-controllers-and-haptics-3243e8399d29. Accessed 2 Sep 2019

[CR14] Lessiter J, Freeman J, Keogh E, Davidoff J (2001). A cross-media presence questionnaire: the ITC-sense of presence inventory. PRESENCE Virt Augment Real.

[CR15] Lewis JR (1995). IBM computer usability satisfaction questionnaires: psychometric evaluation and instructions for use. Int J Human-Comput Interact.

[CR16] Lu F, Davari S, Lisle L, Li Y, Bowman DA (2020) Glanceable AR: evaluating information access methods for head-worn augmented reality. In: 2020 IEEE conference on virtual reality and 3D user interfaces (VR), pp 930–939. IEEE

[CR17] MacKenzie IS, Jusoh S (2001) An evaluation of two input devices for remote pointing. In: Little MR, Nigay L (eds) Engineering for human-computer interaction. EHCI 2001. Lecture Notes in Computer Science, vol 2254. Springer, Berlin. doi:10.1007/3-540-45348-2_21

[CR18] MacKenzie IS, Sellen A, Buxton W (1991) A comparison of input devices in elemental pointing and dragging tasks. In: Proceedings of the ACM conference on human factors in computing systems—CHI '91. ACM , New York, pp 161–166

[CR19] Oculus (2019) First Steps. https://www.oculus.com/experiences/quest/1863547050392688/?locale=en_US. Accessed 19 Aug 2019.

[CR20] Owlchemy Labs (2016a) Job Simulator. https://www.oculus.com/experiences/rift/1069133196442024/?locale=en_US. Accessed 19 Aug 2019

[CR21] Owlchemy Labs (2016b) Job simulator screenshot—Office 02.png. https://commons.wikimedia.org/wiki/File:Job_Simulator_screenshot_-_Office_02.png. Accessed 30 Aug 2019

[CR22] Paas FGWC, van Merriënboer JJG, Adam JJ (1994). Measurement of cognitive load in instructional research. Percept Mot Skills.

[CR23] PlayStation (2018) Europe: Beat Saber 1. 2018 https://www.flickr.com/photos/playstationblogeurope/30807062337/. Original image used under Attribution-NonCommercial 2.0 Generic Creative Commons License. Retrieved 30 Aug 2019

[CR24] Roth D, Latoschik ME (2019) Construction of a validated virtual embodiment questionnaire. arXiv preprint arXiv:1911.10176. 10.1109/TVCG.2020.3023603 32941148

[CR25] Samad M, Gatti E, Hermes A, Benko H, Parise C (2019) Pseudo-haptic weight: changing the perceived weight of virtual objects by manipulating control-display ratio. In: Proceedings of the 2019 CHI conference on human factors in computing systems (CHI '19). ACM, New York, NY, Paper 320, doi:10.1145/3290605.3300550.

[CR26] Sauro J, Dumas JS (2009) Comparison of three one-question, post-task usability questionnaires. In: Proceedings of the conference on human factors in computing systems, pp 1599–1608. doi:10.1145/1518701.1518946.

[CR27] Slater M, Usoh M, Steed A (1995). Taking steps: the influence of a walking technique on presence in virtual reality. ACM Trans Comput-Human Interact.

[CR28] Suchoski JM, Barron A, Wu C, Quek ZF, Keller S, Okamura AM (2016) Comparison of kinesthetic and skin deformation feedback for mass rendering. In: 2016 IEEE international conference on robotics and automation (ICRA). doi: 10.1109/ICRA.2016.7487593

[CR29] Tactical Haptics (2019) https://tacticalhaptics.com/products/. Accessed 2 September 2019.

[CR30] Vannette DL (2019) The qualtrics handbook of question design. https://www.qualtrics.com/ebooks-guides/qualtrics-handbook-of-question-design/. Accessed 2S Sep 2019.

[CR31] Wanderley MM, Orio N (2002). Evaluation of input devices for musical expression: Borrowing tools from HCI. Comput Music J.

[CR32] Whitmire E, Benko H, Holz C, Ofek E, Sinclair M (2018) Haptic revolver: touch, shear, texture, and shape rendering on a reconfigurable virtual reality controller. In: Proceedings of the conference on human factors in computing systems CHI 2018, April 21–26, 2018, Montreal, QC, Canada

[CR33] Willis GB (1999) Cognitive interviewing: A “How To” guide. In surveys short course presented at the 1999 Meeting of the American Statistical Association. https://www.hkr.se/contentassets/9ed7b1b3997e4bf4baa8d4eceed5cd87/gordonwillis.pdf.

[CR34] Witmer BG, Singer MJ (1998). Measuring presence in virtual environments: a presence questionnaire. Presence Teleop Virt.

